# The SAHCS gender-affirming healthcare guideline and the *Southern African Journal of HIV Medicine*: Scientific, ethical and editorial concerns

**DOI:** 10.4102/sajhivmed.v27i1.1830

**Published:** 2026-06-24

**Authors:** Mark E. Patrick

**Affiliations:** 1 Retired Paediatrician, Pietermaritzburg, South Africa

This letter by Mark E. Patrick responds to Tomson et al.^1^ (2021). It is followed by a response from the Editor-in-Chief and Managing Editor of the *Southern African Journal of HIV Medicine*, and the Chief Executive Officer of the Southern African HIV Clinicians Society.

In 2021, the Southern African HIV Clinicians Society (SAHCS) published the *Southern African HIV Clinicians Society Gender-Affirming Healthcare Guideline for South Africa* in this Journal.^1^ On 13 March 2026, the Journal published an editorial expression of concern that the guideline ‘did not undergo the Journal’s standard journal-led external peer-review process’.^2^ The guideline draws on the World Professional Association for Transgender Health (WPATH) *Standards of Care, Version 7 (SOC-7)*, published in 2012.^3^

International developments have raised concerns about the scientific rigour^4^ and ethical foundation of WPATH’s approach.^5^ *The WPATH Files: Pseudoscientific Surgical and Hormonal Experiments on Children, Adolescents, and Vulnerable Adults* offers internal perspectives on WPATH’s practices.^6^ As a result of state-led systematic reviews, the United Kingdom,^7^ Finland,^8^ Sweden,^9^ and a growing number of other countries no longer rely on WPATH and its *Standards of Care* for minors with gender-related distress.

Mirroring WPATH’s *SOC-7*, the SAHCS guideline recommends gender-affirming healthcare (GAHC) for transgender and gender-diverse (TGD) people, including minors.

A reading of the SAHCS guideline raises pressing questions:

- What is the medical definition of a TGD child, adolescent, or young adult?
- What is the epidemiology of being TGD, and how is this understood medically?
- What is the scientific basis for asserting that people of any age can change their sex?

The SAHCS guideline provides us with no medically coherent, evidence-based definition for the term ‘TGD’, nor does it provide us with biologically plausible explanations for the determinants of being TGD, or a robust, evidence-based rationale for its recommendations. The SAHCS guideline asserts that being TGD is an identity; that clients can change sex at any age; and that this must be performed through social, medical and surgical interventions.

The SAHCS guideline details core components of gender-affirming healthcare, including recommendations for:

- Puberty blockers administered off-label to halt normal pubertal development.
- Cross-sex hormones administered off-label to induce opposite sex body morphology.
- Surgical interventions such as breast excision, castration, penile inversion vaginoplasty, and phalloplasty utilising forearm muscle and skin grafts.

For these interventions, the SAHCS guideline advises that:

[*A*] child who is over 12 years of age, … may independently give informed consent to … intervention for GAHC without legally requiring the support or consent of their parents/legal guardians (p. 21).^10^

The irreversible and lifelong consequences of these interventions and the consent process recommended demand rigorous scientific scrutiny and ethical oversight. Because of the enormity of these interventions and their impact on young and vulnerable people, the publication by the SAHCS of such a guideline demands thorough, independent review.

Noting that there was no journal-led external peer-review, that the National Department of Health has not endorsed or approved this guideline, and that the Health Professions Council of South Africa (HPCSA) has issued an advisory on the off-label use of drugs to treat gender dysphoria,^11^ the following questions regarding the guideline need answers from the SAHCS:

- Is it endorsed by the full SAHCS board?
- Has it been endorsed by any independent medical bodies, for example, the South African Paediatric Association?
- Were the SAHCS guideline’s recommendations on social transition, puberty blockers, cross-sex hormones, and surgeries formulated using globally recognised best-practice frameworks for guideline development, such as AGREE II^12^ or GRADE^13^?
- Do the SAHCS guideline’s consent recommendations meet independently assessed medical, ethical, legal, and human rights standards?
- Should patient harm result from using the SAHCS’s guideline, what medico-legal defences exist for individual practitioners, and how will the SAHCS and the Journal support them? (In February 2026, de-transitioner Fox Varian successfully sued her psychologist and plastic surgeon for breast removal at age 16. Casting doubt on his own WPATH guideline, Dr Loren Schechter, plastic surgeon and current WPATH president-elect, a witness for Varian, testified that *SOC-7* should **not** be considered a legal standard of care.^14^)

The SAHCS exists to provide high-quality leadership in HIV care, including for TGD people. The mission of its Journal is to disseminate original research and support high-level learning in HIV medicine.^15^ It is concerning that the SAHCS and its Journal – in publishing this guideline – recommend interventions that cause sterilisation and create lifelong medical dependency.

The SAHCS is a trusted and ‘powerful and independent voice within Southern Africa, with key representation from the most experienced and respected professionals working in the fight against HIV’,^16^ The SAHCS and its Journal should maintain their scientific and ethical integrity by focusing on their core mission.

Owning and publishing a guideline that recommends sex-change interventions in children, adolescents and young adults raises serious questions of patient safety, medical ethics and publication integrity.

The SAHCS guideline did not undergo the standard journal-led external peer-review process. It does not fall within the scope of the Society or its Journal. For reputational and medico-legal reasons, the SAHCS needs to remove this guideline from its distinguished collection of HIV-medicine guidelines.
